# Extensive genotype-phenotype heterogeneity in renal cell carcinoma - a proof-of-concept study

**DOI:** 10.3389/fonc.2025.1551077

**Published:** 2025-04-25

**Authors:** Jakob Wieke, Christina Jurcic, Adam Kaczorowski, Sarah Böning, Martina Kirchner, Constantin Schwab, Viktoria Schütz, Markus Hohenfellner, Anette Duensing, Albrecht Stenzinger, Stefan Duensing

**Affiliations:** ^1^ Molecular Urooncology, Department of Urology, University Hospital Heidelberg, Heidelberg, Germany; ^2^ Institute of Pathology, University Hospital Heidelberg, Heidelberg, Germany; ^3^ Department of Urology, University Hospital Heidelberg, and National Center for Tumor Diseases (NCT), Heidelberg, Germany; ^4^ Precision Oncology of Urological Malignancies, Department of Urology, University Hospital Heidelberg, Heidelberg, Germany

**Keywords:** renal cell carcinoma, tumor heterogeneity, genotype, phenotype, VHL, mTOR, SETD2

## Abstract

**Background:**

Renal cell carcinoma (RCC) is characterized by a high degree of genomic but also functional intratumoral heterogeneity (ITH). Mutations in *VHL*, chromatin remodeling genes such as *SETD2* and genes that regulate the PI3K/AKT/mTOR pathway have been identified as recurrent drivers despite genomic ITH. Whether and to what extent these mutations shape functional ITH including the formation of spatial niches is incompletely understood. Herein, we analyze the correlation between mutational drivers and their functional proxies in a spatially defined manner.

**Methods:**

A total of 23 RCCs were analyzed by panel next-generation sequencing followed by immunohistochemistry for five functional proxies for key genetic alterations including the expression of CD31, GLUT1, phospho-mTOR S2448, H3K36me3 and Ki-67. Antibody stainings were scored semiquantitatively in the tumor periphery and the tumor center.

**Results:**

Unexpectedly, the presence of a *VHL* mutation was not found to correlate with its functional proxies including the expression of CD31/microvessel density or the expression of the glucose transporter GLUT1. Likewise, there was no correlation between the presence of activating mutations in genes of the PI3K/AKT/mTOR pathway and the expression of activated phospho-mTOR S2448. Furthermore, mutations in the methyltransferase gene *SETD2* were not found to correlate with the expression level of its downstream target H3K36me3. Lastly, there was no correlation between the expression of the proliferation marker Ki-67 and the number of driver mutations.

**Conclusion:**

This proof-of-concept study adds genotype-phenotype heterogeneity as additional layer of complexity to the known genomic and functional ITH in RCC.

## Introduction

Renal cell carcinoma (RCC) is among the most lethal urological malignancies once metastatic (1). It is characterized by a high degree of genomic intratumoral heterogeneity (ITH) (2). Despite this general notion, several clonal genomic driver aberrations have been identified and a classification of clear cell RCC (ccRCC) based on the type, number and timing of driver mutations has been proposed (3). Among the high confidence driver genes in ccRCC are *VHL*, which plays a crucial role in oxygen sensing and in counteracting hypoxia through VEGF upregulation and neoangiogenesis, and genes involved in chromatin remodeling, for example, *SETD2*, *PBRM1*, and *BAP1* (4). Moreover, genes that regulate the PI3K/AKT/mTOR pathway such as *PTEN* or *MTOR* itself also function as mutational drivers in ccRCC (4).

Besides genomic ITH, there is compelling evidence for functional ITH, which includes the formation of intratumoral niches that are occupied by tumor cells with certain functional properties (5, 6). The most obvious niches in RCC are the tumor center and the tumor periphery with the latter being a hotspot for proliferation and activation of intracellular signaling pathways (5, 7). Importantly, a previous study could not detect any niche-specific genetic alterations to explain the enhanced tumor cell proliferation in the tumor periphery (5). Instead, there is evidence to suggest that the adjacent tumor stroma plays a role in driving tumor cell proliferation in this niche (8). These findings highlight the important role of non-genetic factors in modulating key tumor characteristics in RCC.

The therapeutic landscape of RCC has evolved significantly over the past decades and the current standard of care involves a combination of immune checkpoint inhibitors and tyrosine kinase inhibitors (9, 10). Interestingly, both of these treatment modalities target primarily the tumor microenvironment (cytotoxic T cells and the tumor vasculature, respectively) rather than the tumor cells. Despite these therapeutic advances, a substantial proportion of patients with advanced RCC will ultimately experience malignant progression and succumb to the disease (11). It is hence paramount to continue to expand the treatment armamentarium and to develop better biomarkers for patient risk stratification (12). One promising avenue may be to use genetic information to personalize treatment decisions. However, there are a number of intricacies that need to be taken into consideration. For example, while mutations in mTOR pathway genes were found in patients with metastatic RCC who benefited from mTOR inhibitors, the majority of responding patients did not harbor mTOR pathway gene mutations (13). A follow-up study showed in fact no correlation between mTOR pathway gene alterations and response to rapalogs (14). These findings raise the general question whether a certain genotype always and inevitably translates into corresponding downstream effects.

In the present proof-of-concept study, we show a substantial disconnection between somatic mutations in RCC driver genes and their expected phenotypic effects (referred to as functional proxies). Our findings establish genotype-phenotype heterogeneity as an additional form of ITH and highlight the challenges of biomarker development and precision medicine in RCC.

## Patients and methods

### Patients

Formalin-fixed, paraffin-embedded (FFPE) tumor samples obtained from a total of 23 consecutive patients with RCC (**Table 1**) were analyzed by targeted next-generation sequencing (NGS) and immunohistochemistry. Tissue samples were obtained from primary tumors (n=15), local recurrences (n=2) or metastatic lesions (n=6). The histology was clear cell (n=17), papillary (n=4), chromophobe (n=1) and collecting duct carcinoma (CDC; n=1). All patients underwent surgery at the Department of Urology of the University Hospital Heidelberg. FFPE sections were provided by the tissue bank of the National Center for Tumor Diseases (NCT) Heidelberg in accordance to the regulations of the tissue bank and after approval by the Ethics Committee of the Medical Faculty Heidelberg of the University of Heidelberg (206/2005, 207/2005, S-864/2019).

**Table 1:** Clinico-pathological patient characteristics.

Patient characteristics	(n=23)
Sex (m/f)	7/16
Age, years (mean)	62.8
TNM stage, n (%)	
pT1	5 (21.7)
pT2	3 (13)
pT3	12 (52.2)
pT4	1 (4.4)
pTx	2 (8.7)
p/cN0	10 (43.5)
p/cN1	4 (17.4)
p/cNx	9 (39.1)
p/cM0	8 (34.8)
p/cM1	9 (39.13)
p/cMx	6 (26.1)
Fuhrman Grade, n (%)	
1	1 (4.4)
2	9 (39.1)
3	6 (26.1)
4	3 (13)
unknown	4 (17.4)
Histology, n (%)	
Clear Cell	17 (73.9)
Papillary	4 (17.4)
Chromophobe	1 (4.4)
CDC	1 (4.1)
Tissue origin, n (%)	
Primary tumor	15 (65.2)
Local recurrence	2 (8.7)
Metastatic lesion	6 (26.1)

### Next-generation sequencing

The NGS analysis of the 23 patients has previously been reported (15). Targeted panel sequencing was performed using the capture-based TruSight™ Oncology 500 panel (Illumina, Cambridge, UK) that covers 523 genes including all relevant RCC driver genes. Mutations were classified as pathogenic, likely pathogenic, activating or likely activating.

### Immunohistochemistry and tissue analysis

FFPE sections were first baked overnight at 37°C. The next day, the slides were deparaffinized in xylene and rehydrated in a graded ethanol series. Antigen retrieval was performed in a steamer using Target Retrieval Solution (Dako, Glostrup, Denmark) followed by quenching with 3% hydrogen peroxide solution and epitope blocking with goat serum. The primary antibodies used were directed against: Ki-67 (Dako, MIB-1, 1:100), phospho-mTOR S2448 (Cell Signaling, Beverly, MA, USA; 49F9, 1: 100), CD31 (Dako, JC70A, 1:100), GLUT1 (Invitrogen, Waltham, MA, USA; SA0377, 1:100), H3K36me3 (Cell Signaling, D5A7, 1:100). Primary antibodies were incubated at 4°C overnight, antibodies against GLUT1 and CD31 were incubated for two nights. The slides were treated with a biotinylated secondary antibody for 3 h at 37°C, followed by the application of POD-streptavidin for 30 minutes. Sections were counterstained with hematoxylin (Sigma-Aldrich, St. Louis, MO, USA) and dehydrated in ethanol before mounting (Histomount, Life Technologies, Frederick, MD, USA).

For each tumor section, we defined the tumor periphery as the outermost zone of the tumor directly adjacent to non-malignant stroma. The tumor center was defined as at least one 20x microscopic field away from the border of the tumor.

For CD31, Ki-67 and phospho-mTOR S2448, representative areas of the tumor periphery and the tumor center were selected and semiquantitatively assessed (median number of areas for CD31, n=5, range 5-10; phospho-mTOR S2448, n=10, range 5-10; Ki-67, n=10, range 4-10). CD31 was scored as microvessel density (MVD) i.e., the number of CD31 positive blood vessel cross-sections per 40x high power field (HPF; 10x ocular lens). The number of Ki-67 positive cells was counted using photomicrographs captured with a 20x objective and a 10x ocular lens (field number 25) thus yielding a field of view (FOV) of 1.23 mm^2^.

For the evaluation of phospho-mTOR S2448 as well as GLUT1 and H3K36me3, we used a modified immunoreactive score (IRS) with staining intensity scored as 0=negative, 0.5=negative-weak, 1=weak, 1.5=weak-moderate, 2=moderate, 2.5=moderate-strong and 3=strong) and the proportion of positive cells scored as 0=negative, 1=<10%, 2 = 10%-50%, 3 = 50%-80%, 4=>80%. All IHC stainings were scored independently by two observers (J.W. and S.D.). To assess the interobserver reliability (IOR) for CD31 and Ki-67 counts, randomly selected tumors were re-counted by one observer and compared to counts by the other observer. The IOR for CD31 with respect to the peak expression in tumor periphery or center was 100%. The IOR for individual counts with a 25% tolerance range was 66.7%. The IOR for Ki-67 with respect to the spatial peak expression was likewise 100%. The IOR for individual counts with a 25% tolerance range was 83.3%.

### Statistical analysis

GraphPad^®^ (Boston, MA, USA) Prism 9 was used for statistical analysis and the nonparametric Mann-Whitney U or Kruskal-Wallis tests were applied. Both are non-parametric tests and hence suitable for all data that do not follow a normal distribution, which is the case for almost all biological data. Categorical variables were analyzed using Fisher’s Exact Probability test (two-tailed). Statistical significance was accepted at p<0.05.

## Results

### Mutational drivers and their functional proxies in RCC

**Figure 1:**
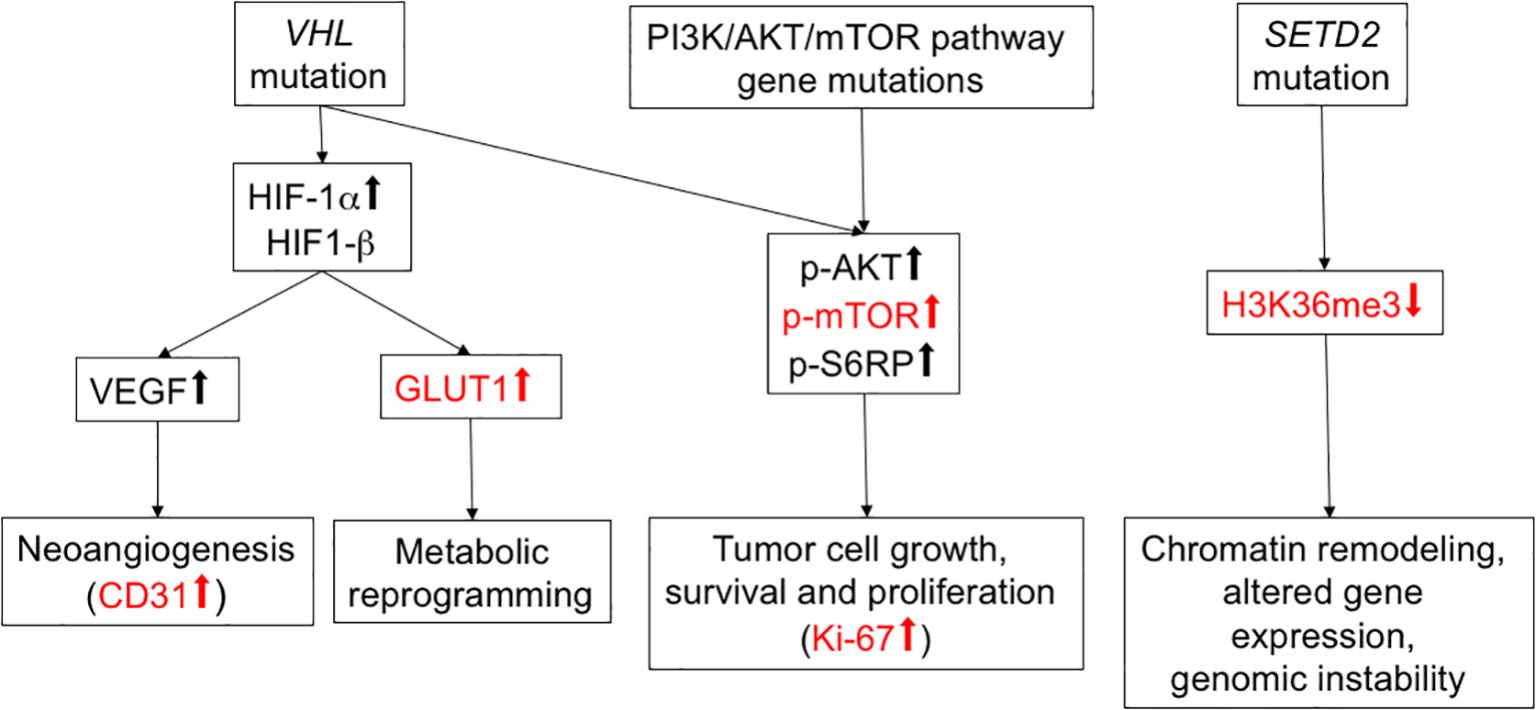
Overview of selected RCC mutational drivers and their downstream pathways. Functional proxies of mutational driver events relevant for the present study are shown in red.

**Figure 2:**
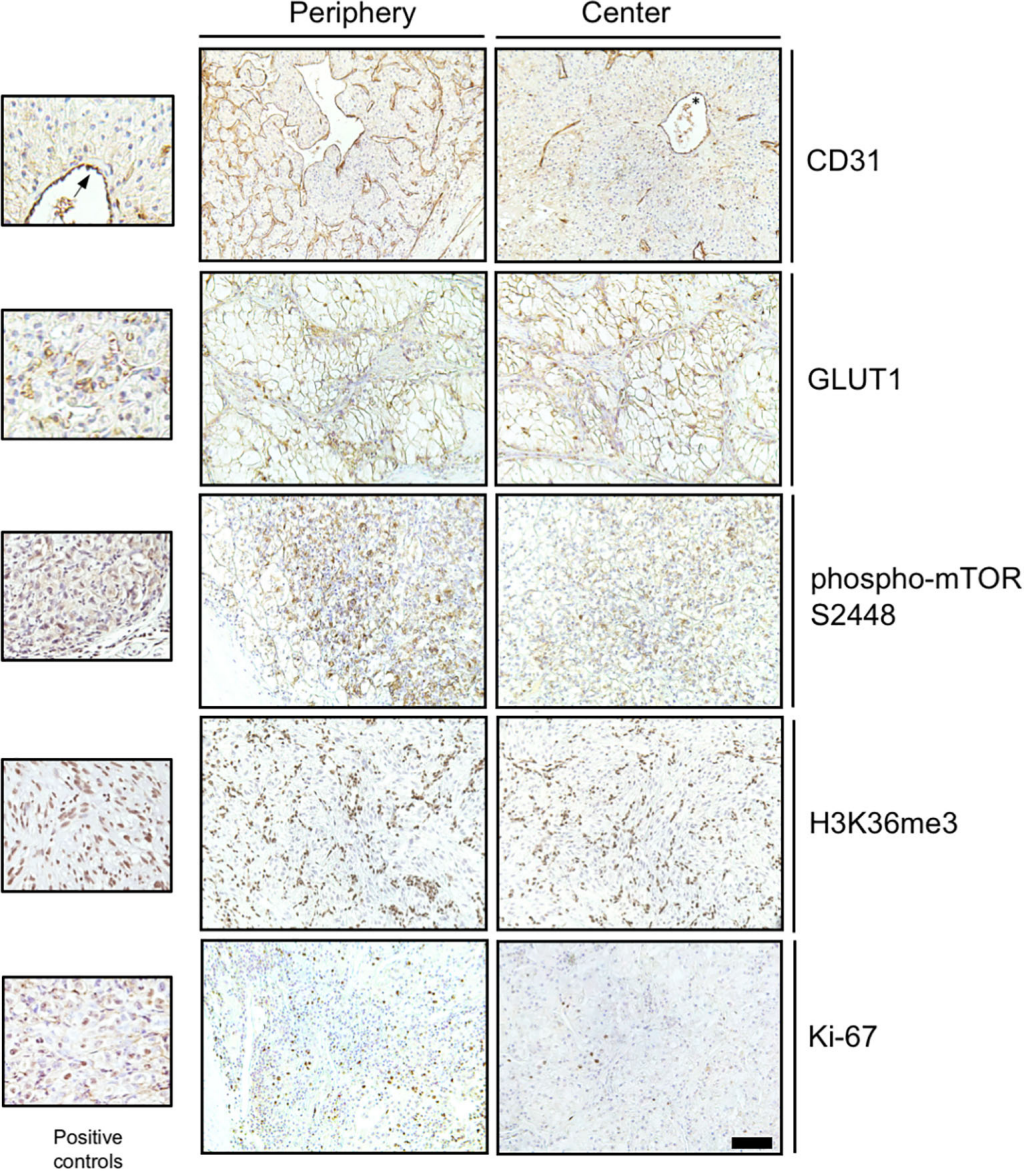
Immunohistochemical detection of functional proxies of mutational driver events in RCC. Representative photomicrographs of immunohistochemical stainings for CD31, GLUT1, phospho-mTOR S2448, H3K36me3 and Ki-67 in the tumor periphery and the tumor center are shown. Positive controls include endothelial cells for CD31 (asterisk and arrow), red blood cells for GLUT1 and Caki-1 RCC cells grown as subcutaneous xenografts (Charles River Laboratories, Freiburg, Germany) for phospho-mTOR S2448, H3K26me3 and Ki-67. Scale bar for large panels = 100 µm.

Frequent mutational driver genes in ccRCC are *VHL*, PI3K/AKT/mTOR pathway genes and *SETD2* (4). The expression of five protein proxies (CD31, GLUT1, phospho-mTOR S2448, Ki-67, H3K36me3; **Figure 1**) was analyzed in the tumor periphery and the tumor center of 15 primary tumors and two local recurrences (**Figure 2**). No distinction between the peripheral and central niche was made in metastatic lesions since fundamentally different growth conditions can be presumed.

**Figure 3:**
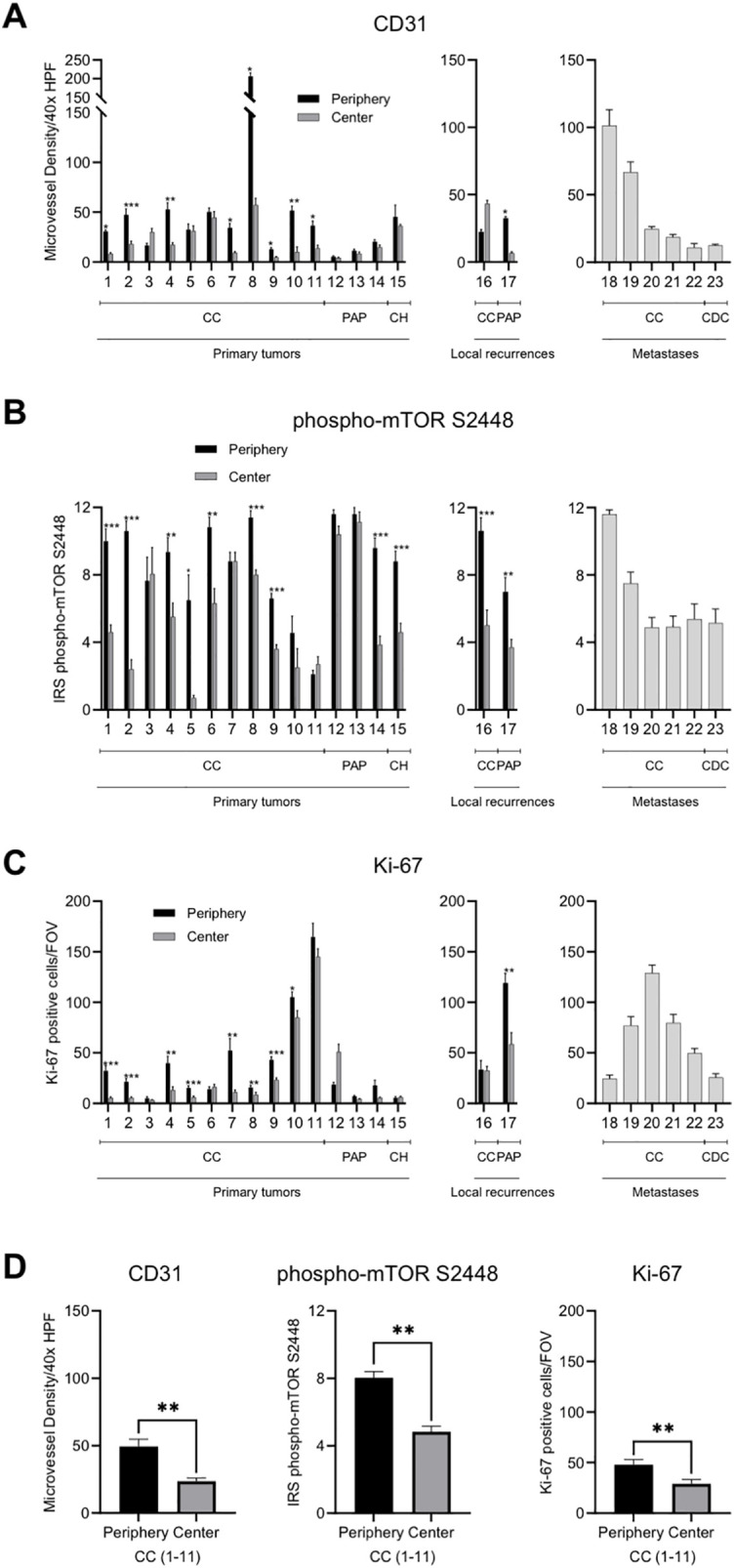
Intratumoral spatial expression of CD31, phospho-mTOR S2448 and Ki-67 in RCC. **(A–C)** Quantification of the expression of CD31, phospho-mTOR S2448 and Ki-67 in a total of 23 primary tumors, local recurrences, and metastases. Tumor periphery and tumor center were assessed separately in primary tumors and local recurrences. Metastases were excluded from the spatial analysis since fundamentally different growth characteristics can be presumed. Each bar represents mean and standard error of at least four and up to a maximum of ten tumor areas. **(D)** Quantification of the average expression of the three functional proxies in 11 primary tumors with clear cell histology subdivided into tumor periphery and tumor center. HPF, high power field; CC, clear cell; PAP, papillary; CH, chromophobe; CDC, collecting duct carcinoma; IRS, immunoreactive score; FOV, field of view [1.23 mm^2^]. Asterisks indicate statistical significance: * p<0.05, **p<0.005, ***p<0.0005.

The expression of CD31 positive blood vessels (scored as MVD) was used as a proxy for *VHL* inactivation (16). Fourteen of the 15 primary tumors showed an increased MVD in the tumor periphery (93.3%; **Figure 3A**). The two local recurrences showed an uneven distribution of the MVD. Metastases showed a considerable intertumoral heterogeneity with a range of 10.8 to 101.4 CD31 positive blood vessels cross-sections/40x HPF.

Phospho-mTOR S2448 staining served as a proxy for mutations in PI3K/AKT/mTOR pathway genes. The expression was consistently higher in the tumor periphery of primary tumors (80%) as well as local recurrences (100%) when compared to the tumor center (**Figure 3B**). Metastatic lesions showed, again, a heterogeneous staining with a minimum mean IRS of 4.9 and a maximum mean IRS of 11.6.

Expression of the proliferation marker Ki-67 followed the same pattern and all primary tumors except tumors 6, 12, and 15 showed an increased tumor cell proliferation in the tumor periphery (80%; **Figure 3C**). One of the two local recurrences also showed this pattern. The tumor cell proliferation rate in metastases ranged considerably with a minimum of 24.4 Ki-67 positive cells/FOV and a maximum of 128.9 Ki-67 positive cells/FOV.

In summary, all three functional proxies of mutational driver events in ccRCC showed differences in the expression between tumor periphery and tumor center with the tumor periphery showing on average a statistically significant enhancement of neovasculature, intracellular signaling pathway activation and tumor cell proliferation (all p<0.001; **Figure 3D**).

**Figure 4:**
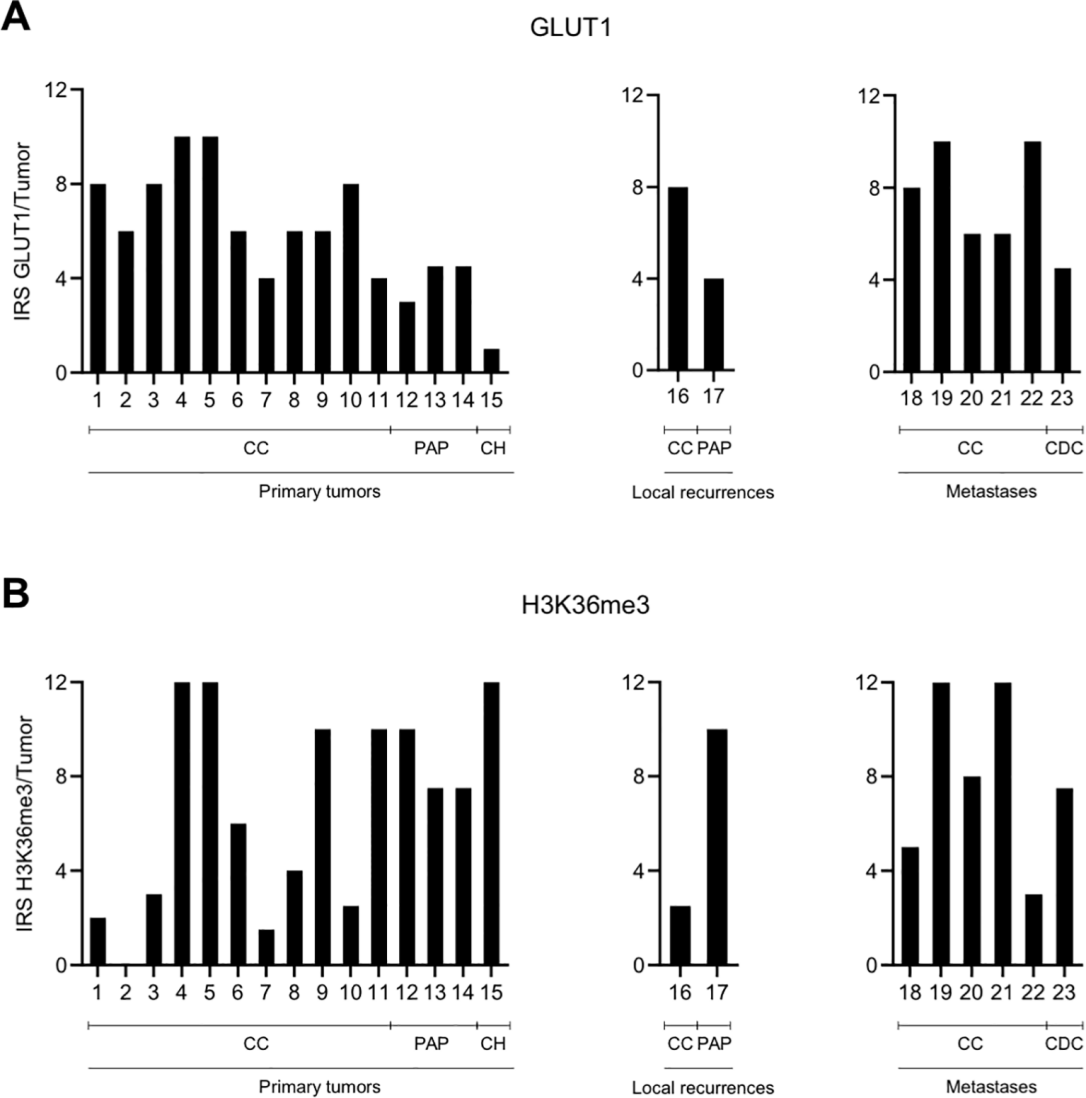
No spatial differences in the intratumoral expression of GLUT1 **(A)** and H3K36me3 **(B)**. Graphic representation of the immunoreactive score (IRS) for GLUT1 and H3K36me3 per tumor. Because of the homogenous expression patterns, no subdivision into tumor periphery and tumor center was performed. CC, clear cell; PAP, papillary; CH, chromophobe; CDC, collecting duct carcinoma.

There were no differences in expression between tumor periphery and tumor center for the functional proxies GLUT1, which is also regulated by the VHL-HIF axis, and H3K36me3, which is methylated by SETD2 (**Figure 4**). In line with previous results (17), GLUT1 showed a higher expression in ccRCC compared to papillary or chromophobe RCC (p<0.05; Mann-Whitney U-test; **Figure 4A**). H3K36me3 was found to show a high degree of intertumoral heterogeneity with a wide range of the IRS between 0 and 12 in primary tumors (**Figure 4B**). The IRS also varied widely among locally recurrent and metastatic RCC (range, 2.5-12; **Figure 4B**).

In summary, these results underscore that tumor periphery and center represent distinct spatial niches in RCC. However, our finding that not all functional proxies followed this pattern suggests that some markers may be more susceptible to external stimuli than others (8).

### No correlation between RCC driver gene mutations and their functional proxies

**Figure 5:**
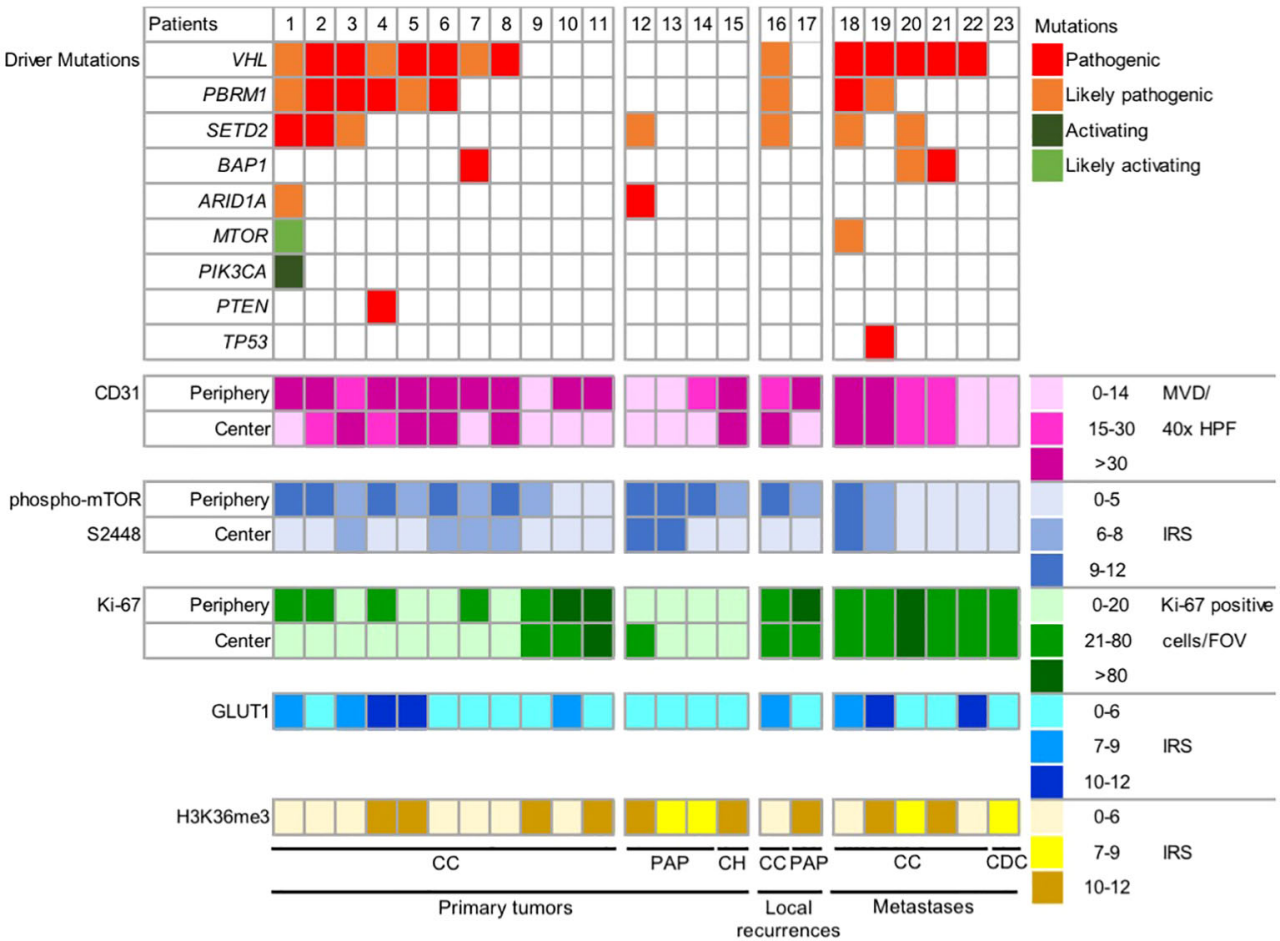
No correlation between mutational drivers and their functional proxies in RCC. Graphic representation of driver mutations and expression of five functional proxies in 23 RCCs. Metastases were excluded from the spatial analysis. Because of the non-heterogeneous expression patterns, no subdivision into tumor periphery and tumor center was performed for GLUT1 and H3K36me3. Color gradients reflect the protein expression levels from low to high. MVD, microvessel density; HPF, high power field, IRS, immunoreactive score; FOV, field of view [1.23 mm^2^]; CC, clear cell; PAP, papillary; CH, chromophobe; CDC, collecting duct carcinoma.

We next sought to determine whether and to what extent the presence of certain ccRCC driver mutations or combinations thereof are represented by their functional proxies (**Figure 5**).

First, we asked whether the presence of a *VHL* mutation (14 of 17 ccRCCs: 82.4%) correlates with in an increased MVD (>30 CD31 positive blood vessel cross sections/40x HPF) or a high GLUT1 expression (IRS 10-12). Only the highest expression regardless of the intratumoral region in which it was found was considered. There was no statistically significant correlation between a *VHL* mutation and the MVD (p=1) or GLUT expression (p=0.541).

Next, we correlated the presence of activating/likely activating mutations in PI3K/AKT/mTOR pathway genes or a pathogenic *PTEN* mutation (two of 17 ccRCCs; 11.8%) to a high expression of phospho-mTOR S2448 (IRS 9-12) and found no statistically significant correlation (p=0.154).

We then correlated the presence of a pathogenic/likely pathogenic mutation in *SETD2* (six of 17 ccRCCs; 35.3%) to a reduced expression of H3K36me3 (IRS 0-6). No statistically significant correlation could be corroborated (p=0.304).

Multiple driver gene alterations defined as *VHL* mutation plus two or more additional mutations in *PBRM1*, *SETD2*, *BAP1* or *PTEN* in analogy to (3) were detected in seven of 17 ccRCC patients (41.2%). Since these tumors have been suggested to show a more aggressive clinical behavior (3), we correlated to the presence of multiple drivers to a high expression of Ki-67 (>80 positive cells/FOV) or moderate to high expression of Ki-67 (>20 positive cells/FOV). No statistically significant correlation could be corroborated with both cut-offs (p=1 and p=0.603, respectively). Remarkably, two of the three tumors with the highest proliferation rate lacked any somatic driver mutations in our panel NGS analysis (patients 10 and 11; **Figure 5**).

Given the heterogeneity of the histological subtypes, a subgroup analysis including only ccRCCs was performed. There was no statistically significant correlation between mutational events and their functional proxies (**Supplementary Figure 1**).

In conclusion, our results suggest that RCC is characterized by an extensive disconnection between mutational drivers and their functional proxies.

## Discussion

Clear cell RCC is characterized by a high degree of genomic and functional ITH (2, 5, 18). Nevertheless, a number of recurrent driver gene mutations have been identified (3, 4). Whether and to what extent these driver gene alterations shape the functional ITH in ccRCC is not known in detail.

In the present report, we made the perplexing discovery that there is no correlation between mutational drivers and their functional proxies. This included MVD and GLUT1 expression in tumors with mutated *VHL*, mTOR phosphorylation in tumors with activating mutations in PI3K/AKT/mTOR pathway genes, H3K36me3 status and mutations in *SETD2* and, lastly, the tumor cell proliferation rate and presence of multiple driver gene mutations.

Clear cell RCCs are highly vascularized tumors and *VHL* loss is crucially involved in this phenotype through hyperstimulation of HIF-dependent transcription of the *VEGF* gene (19). However, VEGF can be upregulated through other mechanisms than *VHL* loss including numerous cytokines, growth factors and hormones (20). Moreover, there is extensive crosstalk between immune cells and endothelial cells. For example, innate immune cells such as tumor-associated macrophages, neutrophils and myeloid-derived tumor suppressor cells secrete pro-angiogenic factors (21). Likewise, T lymphocytes have been reported to secrete VEGF upon stimulation (22). These findings underscore the complexity of neoangiogenesis and may help to explain disconnection between *VHL* status and CD31/MVD in our analysis.

While CD31 expression/MVD was higher in the tumor periphery, the expression of GLUT1 did not show a zonal pattern. Like neoangiogenesis, GLUT1 expression is driven by hypoxia/HIF but also a broad spectrum of additional factors including hormones, growth factors, intracellular signaling pathways such as the PI3K/AKT/mTOR pathway as well as several oncogenes such as *MYC*, *RAS* and *SRC* and the *TP53* tumor suppressor gene (23, 24). Why GLUT1 expression showed no spatial differences when compared to CD31/MVD remains to be determined but one possibility is that neoangiogenesis may be more susceptible to extrinsic stimuli than GLUT1 expression.

Activation of the PI3K/AKT/mTOR pathway not only involves mutations in related pathway genes but also other regulators such as *VHL* itself (25, 26). This signaling pathway is likewise regulated by various extrinsic factors such as growth factors, hormones or inflammatory cytokines (27). Since many of these factors are produced by the adjacent tumor microenvironment, the latter may play an important role in the genotype-phenotype disconnection described herein. The fact that the tumor periphery is a hotspot for the activation of intracellular signaling pathways as shown here and in previous studies (5, 7) lends additional support to this notion.

Our results are in line with previous results showing that H3K36me3 expression is reduced in both *SETD2* mutated and *SETD2* wild-type tumors albeit with a higher frequency in RCCs harboring a *SETD2* mutation (28, 29). In our study, only two patients were found to harbor a pathogenic SETD2 mutation. Although SETD2 exclusively trimethylates H2K36, the levels of H3K36me3 are regulated by other methyltransferases such as SETD3 as well as demethylases of the JHDM family (30). Since the interplay between these “writers” and “erasers” determine H3K36me3 levels, it is obvious that *SETD2* inactivation alone may not necessarily lead to diminished H3K36 methylation. In this context, it is noteworthy that H3K36me3 loss in advanced RCC is much more frequent than *SETD2* mutations thus supporting the notion that alternative factors may contribute to the regulation of H3K36me3 (30).

Three of 17 ccRCCs analyzed showed a proliferation that was in the highest category i.e., >80 Ki-67 positive cells per FOV. Only one of these three tumors belonged to the group of ccRCCs with multiple driver gene mutations (3). The two other ccRCCs showed no detectable driver mutations. Although we cannot formally conclude that the latter ccRCCs were *VHL* wild-type, since no epigenetic analyses were performed, our finding is nonetheless reminiscent of the TRACERx Renal study, were *VHL* wild-type tumors showed a similar proliferation rate compared to tumors with multiple (clonal) driver gene mutations (3).

One can envision various mechanisms through which extrinsic factors such as the tumor microenvironment may overlay mutational events in RCC. It has been reported that cytokines and growth factors secreted by non-malignant cells of the intratumoral and/or extratumoral microenvironment play an important role in this context. For example, FGF-2 secreted by non-malignant cells adjacent to a RCC was found to stimulate RCC cell proliferation (8). Moreover, tumor-derived cytokines have been identified as drivers of intratumoral spatial heterogeneity in RCC (6). Spatial omics and single cell analysis are not only important tools for validating spatial niche formation in RCC, but also instrumental for novel hypothesis-generating approaches to better understand the mechanisms underlying ITH (7, 31–34).

The extensive disconnection between genotype and phenotype is reflected by a number of clinical observations. For example, despite initial evidence, no correlation could ultimately be corroborated between mutations in PI3K/mTOR pathway genes and the response to rapalogs, small molecule inhibitors of mTOR

(13, 14, 35). Genotype-phenotype disconnections as shown herein may contribute to this clinical observation. Furthermore, there was no significant difference in the response to VEGF-targeted agents between patients with inactivated *VHL* and patients with wild-type *VHL* (36). In addition, the response to the VEGFR inhibitors axitinib or pazopanib was found to be independent of the *VHL* status (36). These clinical findings support the notion that RCCs critically depend on certain functional pathways, which is, at least in part, independent of the mutational status of genes involved in these pathway. At the same time, they suggest that a gene-centric view may fall short and prevent the development of more effective therapies for advanced RCC.

Biomarker development in RCC has been particularly challenging. A key implication of our proof-of-concept study is that biomarker development should be multidimensional and very likely needs to entail more than one parameter to be successful. This notion of “biomarker uncertainty” is underscored by reports showing that combined omics approaches can lead to a clinically meaningful substratification of RCC patients (37–39).

Limitations of our study are the relatively small and heterogeneous patient population, the heterogeneity of histological subtypes that limits conclusions on non-ccRCCs, the fact that epigenetic alterations e.g., *VHL* silencing, were not analyzed and that no correlation to treatment responses could be corroborated. The latter is due to the fact that most patients were treated at other academic and non-academic centers following surgery. We were hence unable to retrieve high-quality data on treatment regimens, treatment responses and patient survival. Moreover, we did not attempt to correlate the staining results for functional proxies of mutational events to the actual allele frequency. The reason was that variant allele frequencies are currently not routinely used for clinical-decision making (40).

Validation experiments are currently under way in which digital spatial profiling is used to further corroborate genotype-phenotype discrepancies in RCC (7). This method allows a simultaneous assessment of gene expression in tumors and the surrounding microenvironment and is hence well suited to better understand interactions between these two compartments (41).

Although *in vitro* models for the novel type of tumor heterogeneity reported herein are beyond the scope of the manuscript, further experiments along these lines are clearly warranted. Patient-derived tumor xenografts have been shown to recapitulate the genomic heterogeneity of cancer but extrinsic drivers from the microenvironment including the immune microenvironment are more difficult to emulate (42). Although the same notion may apply to organoids, which are *ex vivo* three-dimensional cell culture models, a number of sophisticated approaches for co-cultivation with immune or stromal cells have been developed (43). The latter may allow to directly dissect the role of certain cell types in driving genotype-phenotype heterogeneity.

Altogether, the present proof-of-concept study adds genotype-phenotype heterogeneity as another layer of complexity to the known genomic and functional ITH in RCC.

## Data Availability

The contributions presented in the study are publicly available. Data can be found here: European Nucleotide Archive (ENA) accession number PRJEB88507.
