# Supplementary figures and images for: Extensive genotype-phenotype heterogeneity in renal cell carcinoma - a proof-of-concept study
Source: Front Oncol. 2025 Apr 25;15:1551077. doi: 10.3389/fonc.2025.1551077 (PMC12061699; doi:10.3389/fonc.2025.1551077)

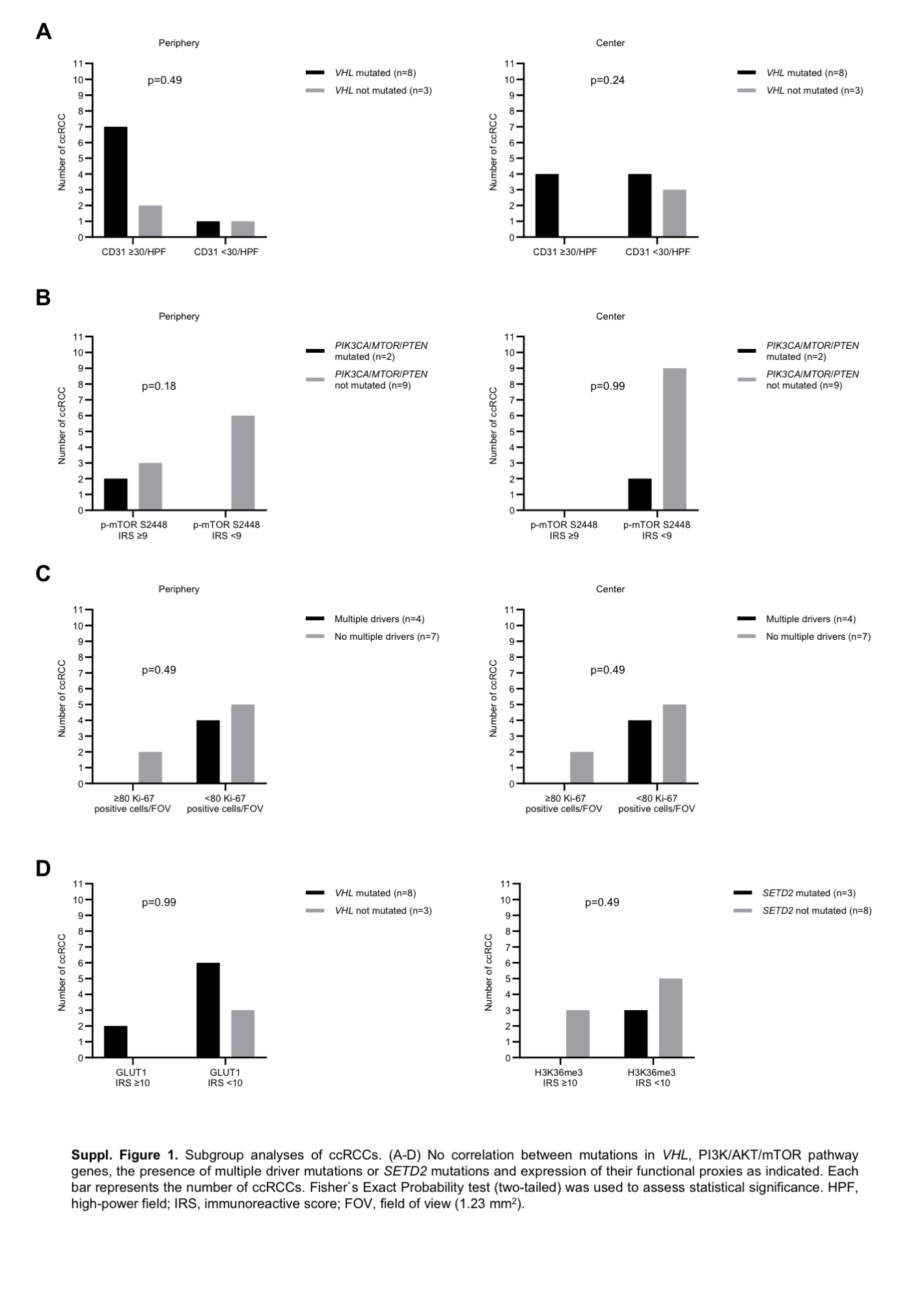

Supplement: Supplementary file 1 [file Image1.jpeg]
